# Psychosocial factors and chronic spontaneous urticaria: a systematic review

**DOI:** 10.1186/s40359-023-01284-2

**Published:** 2023-08-19

**Authors:** Jennifer Donnelly, Katie Ridge, Roisin O’Donovan, Niall Conlon, Pádraic J. Dunne

**Affiliations:** 1 The Centre of Positive Psychology and Health, Royal College of Surgeons, Dublin, Ireland; 2 Clinical and Diagnostic Immunology, St. James’s Hospital, Dublin, Ireland

**Keywords:** Stress, Urticaria, Chronic spontaneous urticaria, Psychosomatic skin conditions, Psychosocial interaction

## Abstract

**Background:**

Psychosocial factors have been informally associated with Chronic Spontaneous Urticaria (CSU); however, the relationship between psychosocial factors and CSU remains relatively unexplored in the scientific literature.

**Objective:**

This review aims to provide an evaluation of peer reviewed studies exploring psychosocial factors and CSU.

**Methods:**

A systematic search was performed over four databases identifying studies exploring psychosocial factors in relation to CSU published between the years 1995 and 2022.

**Results:**

Eighteen studies were included for narrative analysis, and 33 psychosocial factors were identified. These were split into two subgroups: psychosocial factors that were associated with CSU symptoms aggravation/onset (*n* = 20), and psychosocial factors expected to be impacted by CSU symptoms (*n* = 13).

**Conclusion:**

This review has highlighted a need for more research and interventions to support individuals with psychosocial factors involved in CSU.

**Supplementary Information:**

The online version contains supplementary material available at 10.1186/s40359-023-01284-2.

## Introduction

CSU is characterised by the recurrent and unpredictable appearance of wheals (hives), with or without angioedema (swelling), for more than six weeks [1]. CSU is often cited as an autoimmune, mast cell-driven disease. Mast cells degranulate and release histamine [2], resulting in urticaria symptoms. If an obvious trigger (environmental antigen or stimulus) is identified (e.g., food allergy, exercise, heat), then the chronic urticaria is considered inducible (CIindU). CSU on the other hand has no identifiable trigger. As a result, CSU is difficult to treat, with unpredictable remission. Many experiences of CSU last for more than five years, with a person seeking appointments with multiple health professionals before a diagnosis [3]. The first line treatment for CSU is antihistamines at licenced doses. For patients with symptoms that are refractory to high dose antihistamines, treatment is advanced to include the anti-IgE monoclonal antibody omalizumab and cyclosporine [1]; creating a financial burden on healthcare systems, with a variable success rate for disease management [4]. Whilst disease management of chronic urticaria phenotypes is similar, grouping both CSU and CIndU under a chronic urticaria phenotype may obscure important contributors related to these conditions, especially in relation to psychosocial factors. With this in mind, this review aims to examine the experience of CSU specifically, and not conflate results with other types of urticaria. While the pharmacological management of CSU has evolved considerably, measures that might prevent the development of CSU are not apparent [5]. Within non-academic literature and popular sources, stress is often mentioned as a potential trigger to CSU symptoms [6, 7], and understandably so; prolonged stress can increase inflammation, leading to impaired immune function, and increased sensitivity of mast cells [8]. In simple terms, stress that comes from intangible sources (e.g. psychosocial factors) can have a tangible effect on mast cells, and may have a direct effect on CSU symptomology. It is therefore important to assess psychosocial factors within CSU participants, to identify if there are any psychosocial factors affecting this group, leading to symptom onset or aggravation.

The experience of CSU symptoms may also influence psychosocial factors. The uncertainty of causation and duration of the condition can lead patients to feel psychological distress. Furthermore, presence of itch, wheals and/or angioedema can have a profound impact on sleep, social well-being and mental health [9, 10]. In addition, many patients become increasingly anxious about avoiding potential triggers to their symptoms, making unfounded changes to their diet, exercise and lifestyle [11]. It is important to acknowledge that this psychosocial impact may not be applicable to CSU patients in all cultures, due to the limited literature exploring CSU experience in a multi-cultural and global context. Nonetheless, the resultant stress from CSU symptoms can pose a “chicken and egg problem” for researchers when trying to determine if psychosocial factors are a cause, or caused by, CSU symptomatology.

To date, one previous review has explored psychosocial factors and CSU [12]. This review was focused on the psychopathology prevalence in CSU patients, between the dates 1930 to 2012. The authors concluded that even as it was clear that psychosocial factors are indeed affected by CSU symptomatology, research regarding the role of psychosocial factors in CSU onset and aggravation was tenuous. They also noted a lack of research exploring psychosocial interventions for the condition. Despite their efforts, the authors admitted that understanding of psychosocial factors and CSU has advanced in its definition and acceptance within clinical research over the years. They focused heavily on psychopathology throughout their review and included studies with no clear distinction between patients with different types of urticaria, reducing the applicability of results to CSU specifically. It has been 10 years since their publication. A current systematic review of psychosocial factors and CSU is needed to incorporate the more recent understanding of psychosocial factors, and focus specifically on the CSU population.

This review aims to 1) Assess the recent research exploring psychosocial factors and CSU; 2) Provide a narrative analysis of the psychosocial factors associated with CSU; and 3) Identify if any psychosocial interventions have been created to support CSU patients.

## Methods

This systematic review used original studies that address psychosocial factors concerning the aetiology and experience of CSU, and studies proposing psychosocial-based interventions. This review uses the Encyclopaedia of Behavioural Medicine’s definition of psychosocial factors, which includes both individual processes affecting the mind, environmental factors and social structures that affect an individual [13]. To increase the consistency between definitions of CSU, and psychosocial factors, the dates for the study search included studies from 1950 – 2022. Studies were eligible if they met the following inclusion criteria:

### Inclusion criteria

- Original studies using a psychosocial factor as a quantitative or qualitative outcome for CSU/CIU (Chronic Idiopathic Urticaria (CIU) was used interchangeably with CSU terminology in the literature).
- Original studies using a psychosocial intervention for CSU/CIU patients.
- Studies involving adult participants (18 years and above) with a diagnosis of CSU/CIU.

### Exclusion criteria

- Studies without a clearly defined CSU/CIU group, including studies only using the term Chronic Urticaria, or other urticaria types.
- Conference abstracts or short papers with incomplete data.
- Case studies, case reports, or case series studies.
- Reviews, commentaries, or opinion articles.
- Studies without an accessible English translation.

### Search methodology

#### Sources

Studies were found via a systematic search of four databases: MEDLINE, PsycINFO, CINAHL and ProQuest Nursing and Allied Health Source (PNAHS). Whilst CINAHL covers the majority of peer-reviewed papers related to a clinical CSU population, PNAHS holds unique articles not covered in other medical databases [14]. This database includes studies related to alternative and complementary medicine and rehabilitation professions that consider additional psychosocial factors that may be potentially relevant for this review.

#### Search strategy

Search strategies were developed by the reviewer based on the SPIDER framework for mixed methods systematic reviews [15], and finally assessed by a medical librarian. Search strategies for each database combined the concepts of psychosocial factors and Chronic Spontaneous Urticaria using combinations of text words and subject headings. (see Supplementary material 1 for full search strategies). All searches were conducted on April 25, 2022.

#### Data collection and analysis

The systematic review was undertaken in accordance with the PRISMA guidelines [16], and the research protocol was registered on PROSPERO (registration number: CRD42022358259). Results of the initial search were imported into Endnote X9 software to remove duplicate articles. The remaining articles were uploaded to the Covidence online systematic review management system [17] and were independently screened on title and abstract (JD). Articles that were deemed appropriate then progressed to full-text review. Two reviewers (JD, KR) independently assessed full-text articles against eligibility criteria, with any voting conflicts being resolved through discussion. Articles accepted after this process underwent data extraction.

#### Data extraction and synthesis

The data extraction form was created by a reviewer (JD) and piloted (JD, PD) to ensure that the data extraction prompts were understandable, appropriate for the research question, and yielded similar responses from reviewers. Extracted information from the final study set included: author(s), publication year, country of origin, study design, participant characteristics (i.e., mean age, gender), predictor variables (i.e., psychosocial factors), outcome variables (i.e., anxiety measures), and the main findings regarding the relationship between psychosocial factors and CSU were recorded. A narrative synthesis was subsequently conducted based on the review's research question.

#### Quality and risk of bias assessment

Study quality and risk of bias were assessed using the Mixed Methods Appraisal tool (Hong et al., 2018) (See Supplementary material 1). All studies were assessed by two independent reviewers (JD, KR), and any disagreements were resolved through discussion.

## Results

**Fig. 1:**
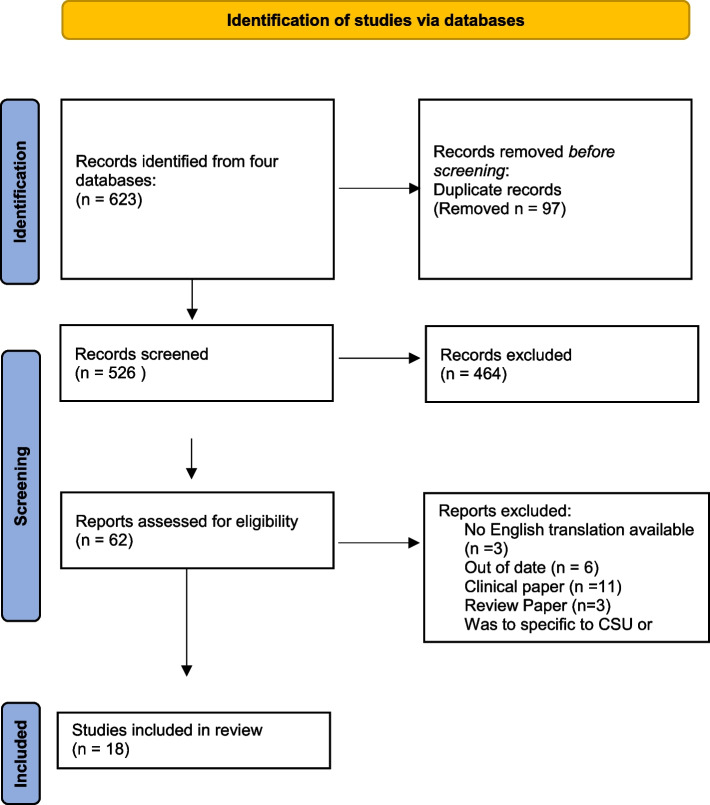
Flow chart of study selection process

Initial database searches yielded 623 records, further reduced to 526 after duplicates were removed. The remaining records were screened on title and abstract, resulting in a further 462 exclusions. A total of 62 articles were assessed against eligibility criteria with 18 studies retained for the final review. Studies were primarily excluded for not separating participants with CSU from other patients with different forms of urticaria. Another common reason for exclusion was that many studies did not adequately focus on psychosocial factors, despite studies typically mentioning stress as a contributing factor of CSU, and CSU having a negative impact on quality of life in their abstracts and introductions. A detailed list of reasons for exclusions at full-text screening is shown in Fig. 1. A summary of included articles is presented in supplementary material 3.

### Study characteristics

After screening with the exclusion and inclusion criteria, 18 studies were approved for data extraction. Demographics comprised mostly female participants (*n* = 2087; 66.31% female, *n* = 1061; 33.71% male). This is in line with research supporting that more women than men are diagnosed with CSU. Regarding the methodology of the 18 studies, most were observational case–control (*n* = 8), and cross-sectional in design (*n* = 5). Two provided a narrative analysis of the experience of CSU diagnosis. Two studies were experimental in design and proposed psychosocial-based interventions. Only one study used a biological measure of stress. See Table 1 for categorisation of study design.

**Table 1:** Categorisation of study design

Total studies for data extraction *n* = 18		
Observational case control studies *n* = 8	Cohort studies *n* = 3	Cross sectional studies *n* = 5
	Experimental study using intervention *n* = 2	Observational narrative Study *n* = 2
	Observational Cohort Studies n = 5	

Studies were conducted in the United Kingdom (*n* = 4), China (*n* = 3), Germany (*n* = 2), Ireland (*n* = 1), Poland (*n* = 1), Italy (*n* = 1), Turkey (*n* = 2), Malaysia (*n* = 1), New Zealand (*n* = 1), Taiwan (*n* = 1) and the United States of America (*n* = 1)

#### Risk of bias

All authors reported no conflicts of interest. Most studies used self-reported questionnaires as a form of quantitative data, which can be prone to response bias, due to participants’ differing perceptions of their wellbeing and personality. Furthermore, the high percentage of cross-sectional studies reduces the possibility of making causal inferences between the psychosocial factors being studied, and CSU symptomatology outcomes. All studies were limited by their control of potential confounds, and three studies were conducted by the same author, using the same participant group. See supplementary material 2 for a summary of quality appraisal and risk of bias using the Mixed Methods Appraisal tool.

### Psychosocial factors correlated with CSU onset and symptom aggravation

**Fig. 2:**
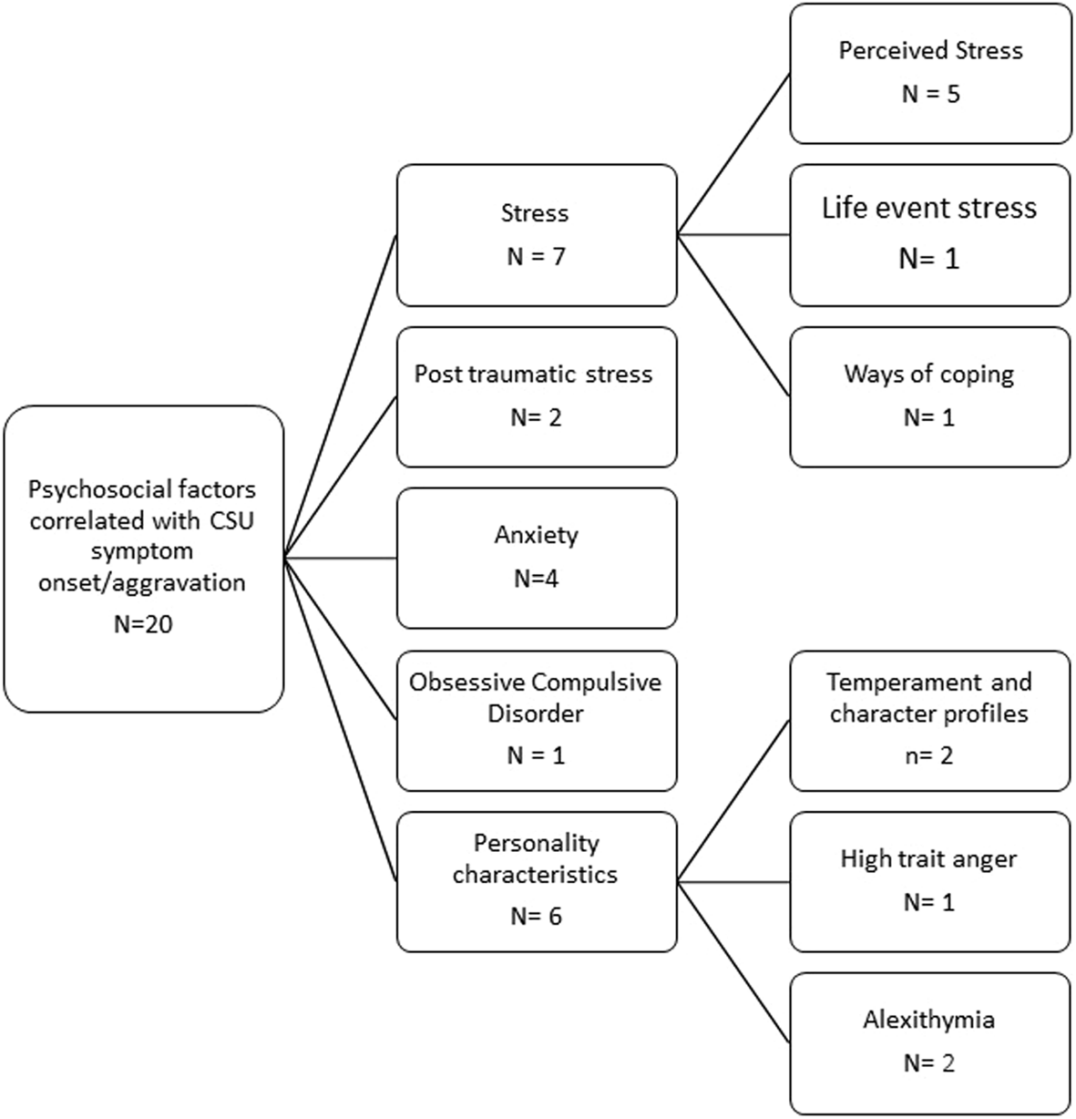
Categorisation of psychosocial factors associated with CSU symptom onset/aggravation

Due to the heterogeneous nature of methodologies and studies, narrative analysis was used to synthesise psychosocial factors and their relationship to CSU. This review has separated the identified psychosocial factors into those suggested to have a causal role in CSU onset/symptom aggravation, and those suggested to be a result of CSU symptoms. Please see Fig. 2 for a categorisation of the psychosocial factors identified.

Narrative analysis of psychosocial factors are as follows:

#### Stress

Ten articles included in this review identified stress as a factor in CSU. Measures of stress included the perceived stress scale (PSS), and the Depression, Anxiety and Stress Scale-21 (DASS-21). In one qualitative interview, participants with CSU identified stress as a “trigger” for their symptoms [18]. One study used biological outcome measures of stress that included a basophil activation test as well as a measure of serum cortisol concentration assessment [19]. One study explored stress as an accumulative process, and used the major life events survey, to find a trend of CSU patients experiencing more self-reported stressful life events than controls. Ways of coping with stress were also measured in a study by Yang, 2005 [20] using the ways of coping checklist, and the major life events survey. Their results indicated that patients with CSU had experienced a stressful life event before the onset of CSU, and were more likely to use negative coping to manage stressful events when compared to controls diagnosed with tinea pedis (a fungal skin condition).

#### Post Traumatic Stress Disorder (PTSD)

Four studies explored PTSD in relation to CSU. These studies utilised the post-traumatic diagnostic scale or were diagnosed through an interview with Diagnostic Short Interview for Mental Disorders (Mini-dips) [21]. Results suggested that CSU patients were more likely to have a diagnosis of PSTD when compared to healthy controls, or allergy patients. It was also suggested that PTSD influenced coping styles, which in turn affected CSU severity [22]. Furthermore, the study by Hunkin and colleagues found that PTSD was co-morbid with alexithymia traits, and correlated with defensive coping mechanisms [23]). Their study also found higher rates of self-reported sleep disturbance and family relationships before CSU onset; however, the measurements used for this study were adapted and modified, which reduces the standardisation of their results when compared to other studies.

#### Anxiety

Anxiety was measured using standardised questionnaires in seven studies. Anxiety was considered as a result of CSU symptomatology and the unpredictability of the condition. However, the study by Hunkin [23] found that anxiety as a character trait was significantly higher in individuals with CSU compared to healthy controls, which indicated that a pre-disposition to anxiety might be a potential trigger of CSU symptoms. However, this was only their interpretation of their results and further research is required. The DASS was also used in a multi-ethnic Asian population [24], which found depression and anxiety to be present in the CSU population; however, this was also mediated by the severity of their disease. One study used the Turkish Version of the Fear of COVID-19 scale (FCV-19S) [25], in addition to the DASS, to investigate how COVID-19- related anxiety impacted CSU symptomatology. Their results further supported the connection between CSU and anxiety, as high levels of fear and anxiety around COVID-19 were associated with increased CSU symptomology.

#### Obsessive Compulsive Disorder (OCD)

In a study of mental health co-morbidities, using the mini-dips interview, it was suggested that only 2% of the CSU population experienced OCD [9]. However, they did not compare this with any control group, and simply found this to add to the prevalence of mental disorders in their CSU patient group. There were no other studies exploring OCD prevalence in CSU patients.

#### Personality characteristics

Personality characteristics were explored in relation to CSU symptom onset using the Neuroticism-Extraversion-Openness Five-Factor Inventory (NEO-FFI) [26] and Temperament and Character Inventory (TCI) [27]. The results from the NEO-FFI indicated higher scores in neuroticism; however, this was only significant for CSU patients with a diagnosis of PTSD. The TCI showed stronger trends of lower cooperativeness, reward dependence and self-directedness when compared to healthy controls. Whereas these personality characteristics are similar, it is hard to compare the two, as different measurements of personality were used. One study also investigated alexithymia using the Toronto Alexithymia Scale (TAS), suggesting that the occurrence of alexithymia was significantly higher in CSU and psoriasis patients when compared to healthy controls. CSU patients scored higher on alexithymia subscales. Other character traits where measured using the STAXI (State-Trait Anger Expression Inventory) [28]. CSU patients had significantly increased state anger as measured by the STAXI when compared with healthy controls and psoriasis patients [29].

### Psychosocial factors negatively impacted by CSU symptoms

**Fig. 3:**
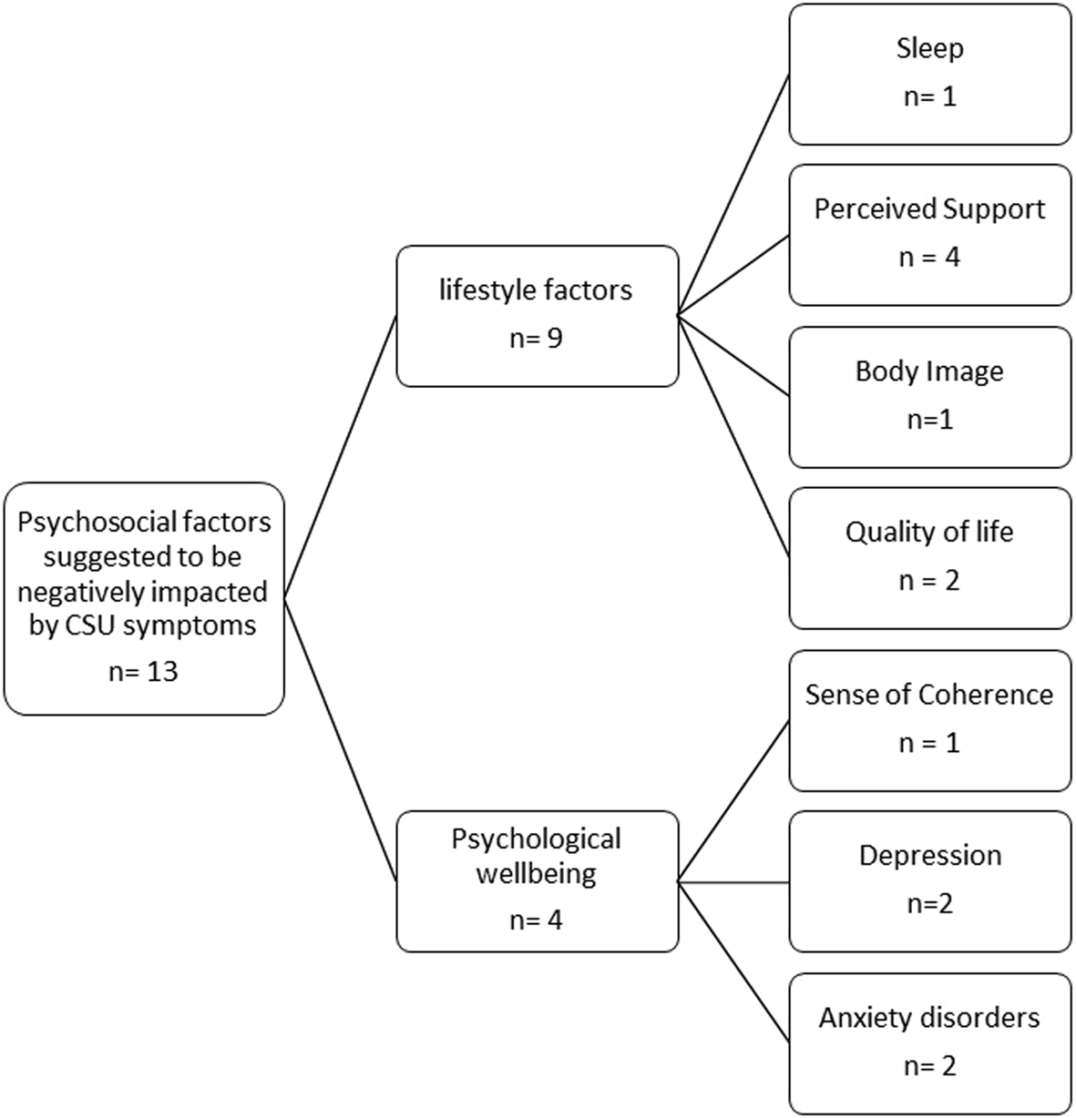
Categorisation of psychosocial factors negatively impacted by CSU symptoms

Within the included studies, psychosocial factors were suggested to be affected by CSU symptoms (*n* = 15). Please see Fig. 3 for an illustration of identified psychosocial factors.

A narrative synthesis of psychosocial factors is as follows:

#### Lifestyle factors

Four studies included a measure involving lifestyle factors. The Dermatology Life Quality Index (DLQI) was used in two studies. Whilst other measures of lifestyle factors included the PERMA profiler (positive psychology-based wellbeing assessment) and a non-standardised questionnaire on sleep physical activity and social support. Quality of life was measured by the DLQI in two studies. A 2022 study by Yong and colleagues [24] found that 36.5% of 115 patients with CSU reported significantly impaired quality of life, correlating with the severity of CSU. Furthermore, 54.8% of their patients also had anxiety when measured by the DASS-21 scale. Another study used DLQI in an epidemiological study in China, finding self-reported overall quality of life to be lower in CSU compared to physical urticaria, and other urticaria types [30].

#### Psychological wellbeing

Six studies included a measure of depression and anxiety disorders, as well as a sense of coherence. DASS-21was employed in two studies, as previously mentioned. The Hospital Anxiety and depression scale was also used. Results indicated that CSU patients had higher levels of depression and anxiety, however there were no significant differences when CSU patients were compared with CIndU controls. Sense of Coherence was suggested to be worse for patients with CSU patients who experienced CSU with angioedema compared to those without.

### Other measures

Two studies incorporated the Symptom Check-List-90-Revised (SCL-90R) [31]. This questionnaire involves nine dimensions or factors reflecting various types of psychopathologies. Both studies reported higher somatic symptoms in patients with CSU when compared to controls [9, 29]. One other study used the Psychosomatic Symptoms Inventory, also suggesting trends in psychosomatic symptoms being associated with stress for CSU participants [20].

#### Qualitative research

Perceived support was identified as a common theme in qualitative interviews exploring patient experience both in Italy [32] and the US [33]. Similar topics were found in all studies. These were described as the patient’s frustration with the care available for CSU, and poor relationships with family members. In the US-based narrative, the patients also reflected a lack of acknowledgement of the emotional impact of their condition from their physicians and the feeling of being “an experiment” due to the uncertainty of their condition. In one study from 2007 [34] 76% of participants reported that their body image was negatively affected by their CSU.

#### Interventions

The previous systematic review stated that researchers should aim to develop interventions to address psychosocial factors and CSU. The literature review found two studies proposing interventions. One study took place in 2015 [35], providing what the authors describe as a “10-week, non-dualistic, psychologically-oriented, mind and body intervention”. The other took place in Ireland and provided an 8-week Attention Based Training (ABT) programme, which involved the use of mantra-based meditation, and educational components on acceptance and compassion [18]. Both were pilot studies. However, the results of these interventions were promising, with participants having a reduction in urticaria symptomatology after each programme [35, 18].

## Discussion

Do psychosocial factors cause/trigger CSU symptoms, or do CSU symptoms have a detrimental impact on psychosocial factors? To address this, we divided the identified psychosocial factors, based on their proposed causality. The most common psychosocial factor associated with CSU onset/symptom aggravation was stress, however there is little agreement between researchers on how stress is best measured, and how to use objective biomarkers to support subjective psychometrics. Personality characteristics related to ways of coping and the expression of anger was also associated with CSU. Poor coping strategies and anger suppression, and subsequent outbursts of emotion may also result in increased stress and inflammation [36, 37]. Our findings suggest that there is a trend towards certain personality types being more predisposed to living stressful lifestyles or suppressing their emotions among CSU populations. This supports previous research indicating that mental stress can manifest as physical symptoms in the body. We also found that perceived support and psychological well-being related to mood and anxiety disorders were negatively impacted by CSU symptoms. However, these outcomes were not specific to CSU patients and were similar to those experienced by individuals with other types of urticaria. Nevertheless, it is still important to consider these factors when exploring interventions for this condition.

Despite the growing interest in the role of psychosocial factors in chronic spontaneous urticaria, there has been a lack of updated reviews on this topic in the past decade, highlighting the need for a more current and comprehensive review. 18 articles were identified that specifically investigated these factors in CSU patients. Despite the recognized importance of psychosocial factors in this condition, their multi-faceted and complex nature makes them challenging to quantify and separate from confounding variables, which is reflected in the quality and reliability of the studies available. Most of the included articles relied on self-reported questionnaires as their main outcome measures, with small participant sizes and limited use of objective measurements, such as biomarkers, to support psychometrics. Despite these limitations, self-reported results provide important insight into the complex role of psychosocial factors in CSU.

In recent years, there has been a growing recognition of the impact of mental stressors and psychosocial factors on physiological functioning. However, studies investigating the "brain-skin" connection in CSU are still lacking, and it is hoped that future studies will combine both physical and psychosocial measures to create a stronger evidence base. One study included in this review used blood cortisol to measure stress in CSU patients and found no significant difference in baseline cortisol concentrations compared to healthy controls, in contrast to previous literature suggesting higher stress levels in CSU patients. However, the authors suggested that these results indicated abnormal function of the hypothalamus, pituitary gland, and adrenal glands (HPA axis) in CSU patients, indicating a heightened response to stressful stimuli. Thus, the results highlight the need for both objective biological measures and subjective psychosocial measures to gain a better understanding of the complex interaction between stress and CSU symptoms.

Whilst these key issues are present in the literature, the current systematic review has identified a trend of high prevalence of psychosocial co-morbidities in CSU patients. Overall, CSU symptoms are suggested to have a detrimental impact on quality of life and mental well-being, and perceived stress may have a causational role in CSU symptoms.

### Comparison with the results of previous reviews

The previous systematic review on psychosocial factors and CSU [12] concluded that more research was needed, with a focus on randomised control trials and interventions. An addition of 11 studies were found that had been published since their initial review. No randomised control trials were identified, and only 2 of these studies proposed psychosocial interventions for this client group; both of which were pilot studies and need further trials to determine their effectiveness for people with CSU. This current review also used a wider definition of psychosocial factors, finding studies incorporating social support, sleep quality and quality of life.

### Strengths

The strengths of this review included crosschecking of data abstraction and screening. The population of this review focused solely on adults aged 18 and over, with a diagnosis of Chronic Spontaneous Urticaria, to allow for the extrapolation of findings to this specific clinical group. This review also included psychosocial factors as a general concept instead of focussing on a specific factor (e.g. stress), as literature on this area is limited, and the authors aimed to provide a narrative summary of the research available, to determine with factors are most relevant to CSU patients.

### Limitations

In their earlier systematic review, Ben-Shosan et al. [12] highlighted that the diagnosis and clinical understandings of both CSU and psychosocial factors have changed over time, and therefore this review only focused on studies published after 1995, to lessen variability in the clinical definition. The studies included in this review contained participants from the UK, European, or East-Asian countries. Consequently, a publication bias might exist in the current literature regarding patients with CSU from other demographics.

### Directions for future research

Future studies should aim to add to research surrounding psychosocial factors and CSU. Researchers should be encouraged to examine the causational relationship between psychosocial factors and CSU symptomatology. Attention should be given to the way CSU patients experience stress. It is important for interventions to be developed for this patient group, to find ways of supporting CSU patients with the experience of stress as a preventative measure, to compliment pharmaceutical approaches, and reduce medical burden. In addition, research should also aim to address the psychosocial impact of CSU on patients' lives, with a particular focus on sleep quality, and anxiety/stress management. Qualitative research has helped highlight areas of wellbeing that are missed in generic questionnaires, however there is a lack of follow up within literature regarding body image, and perceived support for CSU patients; more research in these areas is recommended. It is also encouraged that future research aims to address a lack of randomised control trials regarding psychosocial factors and CSU.

## Conclusion

In summary, this systematic review has highlighted significant gaps in the peer-reviewed literature regarding the relationship between psychosocial factors and CSU. Currently, the wide variety of psychometric tools and methodologies limits the possibility of conducting quantitative analyses of studies. As a result, a narrative comparison of specific psychosocial factors was deemed the most suitable analysis for this review. Existing studies indicate that CSU patients experience higher rates of psychosocial co-morbidities, with stress emerging as the most strongly associated factor with symptom aggravation and onset. Furthermore, the review has identified perceived support and anxiety disorders as the most impacted psychosocial factors by CSU symptoms. Although two psychosocial interventions have been proposed for CSU patients, the small sample sizes in these studies highlight the need for further development and assessment of these interventions. This review underscores the importance of investigating psychosocial factors both as a cause and a result of CSU to improve patient outcomes and quality of life. In addition, it is suggested that future studies consider adopting similar methodologies and consistent psychometrics, to allow for more robust comparison between studies. It is hoped that this summary of previous literature will inspire future research directions, leading to a better understanding of the complex interplay between psychosocial factors and CSU, and more effective treatments for patients and their clinicians.

### Supplementary Information

**Additional file 1: Appendix A. **Supplementary Material 1. **Appendix C. **Supplementary Material 3. **Appendix B.** Supplementary Material 2.

## Data Availability

The data and materials used in this systematic review are available to other researchers upon request. The studies included in this systematic review were identified through a search of databases, including MEDLINE, PsycINFO, CINAHL and ProQuest Nursing and Allied Health Source (PNAHS). The search strategy, keywords and search terms are provided in the Supplementary Material. The full list of included studies, along with their bibliographic details and relevant data extracted for analysis, is available in Appendix C, table 3 of the supplementary material. The complete list of references, is provided in the References section of this article.
