# Supplementary material for: Psychosocial factors and chronic spontaneous urticaria: a systematic review
Source: BMC Psychol. 2023 Aug 19;11:239. doi: 10.1186/s40359-023-01284-2 (PMC10440028; doi:10.1186/s40359-023-01284-2)
Supplement: Supplementary file 1 — Additional file 1: Appendix A. Supplementary Material 1. Appendix C. Supplementary Material 3. Appendix B. Supplementary Material 2. [file 40359_2023_1284_MOESM1_ESM.zip › Appendices.docx]

**Appendices**

**Appendix A, Supplementary Material 1:**

*Academic Search String used for all databases.*

AB(“Chronic Spontaneous Urticaria” OR “CSU” OR “urticaria” or “Chronic Urticaria”) AND AB(“psychosomatic” OR “psycho-somatic” OR “psychosocial” OR “psycho-social” OR “Stress*” OR “Lifestyle” OR “emotional* triggers*” OR “emotion*” OR “Allostatic load” OR “alcohol*” OR “tobacco” OR “substance misuse” OR “adverse life experience*” OR “Adversity”

| Database | Person |  | Outcome | Search Results |
| --- | --- | --- | --- | --- |
|  | “Chronic Spontaneous Urticaria” OR “CSU” OR “urticaria” or Chronic Urticaria” | AND | “psychosomatic” OR “psycho-somatic” OR “psychosocial” OR “psycho-social” OR “Stress*” OR “Lifestyle” OR “emotional* triggers*” OR “emotion*” OR “Allostatic load” OR “alcohol*” OR “tobacco” OR “substance misuse” OR “adverse life experience*” OR “Adversity” |  |
| Medline | 20,448 |  | 2,140,145 | 430 |
| Psychinfo | 333 |  | 773,930 | 92 |
| CINAHL | 1461 |  | 409,784 | 63 |
| ProQuest nursing and allied health source | 876 |  | 157.904 | 38 |
| Total |  |  |  | 623 |

**Appendix B, Supplementary Material 2:**

Table providing an overview of quality assessment of included studies using the Mixed Methods Appraisal Tool. (table will be attached in additional file)

**Appendix C, Supplementary Material 3:**

Table providing an overview of studies used in the narrative analysis, including a brief summary of study results and psychosocial measurements used.

| Title | Main Author | Date | N= Male vs Female participants with CSU | Brief summary of results | Measurements Related to Psychosocial Factors used in Study |
| --- | --- | --- | --- | --- | --- |
| Chronic urticaria in Chinese population: a hospital-based multicenter epidemiological study | Zhong 2014 | 2011 | M, 732: F, 1113 | 14% of CSU patients self- reported emotional disorders | Urticaria Activity score. The Dermatology Life Quality Index (DLQI) |
| Psychological well-being, quality of life and patient satisfaction among adults with chronic spontaneous urticaria in a multi-ethnic Asian population | Yong 2022 | 2022 | M, 33: F, 82 | Severity of CSU was associated with psychological burden | Dermatology Life Quality Index (DLQI), Short Form of Depression Anxiety Stress Scales (DASS-21), Short Assessment of Patient Satisfaction (SAPS) |
| Stress, insomnia, and chronic idiopathic urticaria--a case-control study | Yang 2005 | 2004 | M, 22: F, 53 | Higher scores of somatic and psychosomatic symptoms in CSU population, suggestion of an association between stress and the development of somatic symptoms | Major Life Events Survey, Somatic Symptoms Checklist, Insomnia Scale, Irregularity Index of Daily Life, Ego-function Index, Family Support Index,and Psychosomatic Symptoms Inventory, Ways of Coping Checklist |
| High prevalence of mental disorders and emotional distress in patients with chronic spontaneous urticaria | Staubach 2011 | 2011 | M, 31: F, 69 | Forty-eight of the 100 patients with CSU were found to have one or more mental disorder as assessed by diagnostic interviews and mini-DIPS. The most common mental disorders were anxiety disorders (30%), followed by depressive and somatoform disorders (17% each), adjustment disorder (4%), post-traumatic stress disorder, harmful use of alcohol (3% each), hypochondria, obsessive compulsive disorder (2% each) and alcohol dependency, multiple substance abuse (1% each). Agoraphobia was found to be the most frequent anxiety disorder in patients with CSU (15%;). | mental assessment by one to three extensive diagnostic interviews, and by Mini-DIPS. Hospital Anxiety and Depression Scale German version (HADS), Symptom Check List (SCL-90R) for somatization Global Severity Index of psychological distress (GSI) |
| Feasibility assessment of an 8-week attention-based training programme in the management of chronic spontaneous urticaria | Ridge 2021 | 2021 | M, 3: F, 9 | ABT was acceptable to patients with CSU | Depression and Anxiety Stress Scale (DASS 21), PERMA profiler, Five Facets of Mindfulness Questionnaire(FFMQ), |
| Treating chronic spontaneous urticaria using a brief 'whole person' treatment approach: a proof-of-concept study | Lindsay 2015 | 2015 | M, 1: F, 9 | Three of four had improved urticaria symptomatology | N/A |
| Chronic idiopathic urticaria, psychological co-morbidity and posttraumatic stress: The impact of alexithymia and repression | Hunkin 2012 | 2012 | M, 14: F, 75 | Patients with CIU were more likely to report at least one stressful life event  Psychological comorbidity was higher in patients with CIU | The General Health Questionnaire-12 (GHQ-12) is the short form of a screening device for identifying the likelihood of meeting criteria for a psychiatric disorder. The cut-off score used was 11.The Perceived Stress Scale (PSS) The Posttraumatic Stress Diagnostic Scale (PDS) , The Toronto Alexithymia Scale (TAS-20), The Bendig version of the Taylor Manifest Anxiety Scale (TMAS) |
| Association of Chronic Spontaneous Urticaria With Anxiety and Depression in Adolescents: A Mediation Analysis | Huang 2021 | 2021 | M, 188: F, 205 | CSU was significantly associated with both anxiety and depression. Itching and sleep quality mediated 65.4 and 77.6% of CSU's effects on anxiety and depression, respectively, and CSU had no significant direct effect on anxiety or depression in the mediation models. | A web-based questionnaire survey within 1 day. The questionnaire was self-reported. The questionnaire was comprised of demographic information, history of diseases that might be associated with skin health, history of allergy, cigarette smoking, alcohol drinking, intake of soft drinks, water intake, food taste preference, defecation, sport, sleep quality, anxiety, depression, bath habit, skincare, and sun exposure. Anxiety and depression were measured by the two-item Generalized Anxiety Disorder Scale (GAD-2) and two-item Patient Health Questionnaire (PHQ-2), respectively. Sleep quality was measured by the Pittsburgh Sleep Quality Index (PSQI). |
| Perspectives on Living with Chronic Spontaneous Urticaria: From Onset through Diagnosis and Disease Management in the US | Goldstein 2019 | 2019 | M, 6: F, 19 | Themes associated with living with spontaneous Urticaria: What the Heck is going on?, Living with the Skinemy, Relief and confusion with diagnosis, my own personal hell, I feel like an experiment.   Treatment and Management Themes:   Challenges of the care pathway, The lack of CSU education resources, The challenges of CSU symptom management. | Semi structured interview with patients and separate interviews with physicians  Patient diaries |
| Effect of stress on basophil function in chronic idiopathic urticaria | Dyke 2007 | 2007 | Not reported | There was no significant difference between numbers of CSU patients and normal controls responding to CFR, ACTH or cortisol. However, the responses in the CIU patients were stronger than those in normal controls. There was also a trend towards higher serum cortisol concentrations in CIU patients. The basophil response to CRF and ACTH correlated with the serum cortisol concentration in normal controls, but not in CIU patients. | Basophil activation test  Serum cortisol |
| Relationship between anger and pruritus perception in patients with chronic idiopathic urticaria and psoriasis | Conrad 2008 | 2008 | M, 9: F, 34 | CSU patients have higher alexithymia when compared with psoriasis patients. Both patients with CSU and psoriasis had higher scores related to depression, anxiety and distress  CIU and psoriasis had higher trait anger | Symptom checklist 90R (SCL90R), 21 toronto alexithymia scale, State trail anger and depression inventory |
| The relationship between posttraumatic stress disorder, psychiatric comorbidity, and personality traits among patients with chronic idiopathic urticaria | Chung 2010 | 2010 | M, 18: F, 82 | No. of patients meeting PTSD diagnosis was higher in patients with CSU versus control group  CSU patients with PTSD experienced more anxiety than patients without PTSD | PDS, General Health Questionnaire-2 (GHQ28), the Social Readjustment Rating Scale (SRRS)  PSS, NEO Five Factor Inventory (NEO FFI) |
| Stress, psychiatric co-morbidity and coping in patients with chronic idiopathic urticaria | Chung 2010 | 2010 | Same sample as above | Patients with CSU had significantly higher levels of stress than patients with allergy | General Health Questionnaire-28 (GHQ-28), the Social Readjustment Rating Scale (SRRS), the Perceived Stress Scale (PSS) and the Ways of Coping Checklist (WOC) |
| Posttraumatic Stress Disorder and Chronic Idiopathic Urticaria: The Role of Coping and Personality | Chung 2019 | 2019 | Same Sample as above |  |  |
| Living with Chronic Spontaneous Urticaria in Italy: A Narrative Medicine Project to Improve the Pathway of Patient Care | Cappuccio 2017 | 2015 | Not given | Multiple - poor trust in practitioners, ongoing physical impact of the disease | Narrative only, no quantitative measures |
| Psychological burden of COVID-19 on mild and moderate chronic spontaneous urticaria | Beyaz 2021 | 2021 | M, 135: F, 374 | High levels of fear, depression and anxiety were associated with CSU symptomatology | Turkish version of the Depression Anxiety and Stress Scale 21 (DASS - 21).  Turkish Version of the Fear of Covid 19 scale (FCV-19S) |
| Angioedema Coexisting Chronic Spontaneous Urticaria Negatively Influences Patients' Sense of Coherence, What Results in Susceptibility to Anxiety Symptoms Occurrence | Badura-Brzoza 2021 | 2021 | M, 26: F, 45 | Coexistence of angioedema for CSU patients impairs SOC, particularly in the domains related to meaningfulness, and favours anxiety occurrence. | Urticaria Activity Score (UAS); State-Trait Anxiety Inventory (STAI); Sense of coherence (SOC)-29 |
| Temperament and character profiles of patients with chronic idiopathic urticaria | Alan 2015 | 2015 | M,18: F,52 | The CIU group had significantly higher scores of novelty seeking and lower scores of cooperativeness, reward dependence and self-directedness than the control group. | Visual analogue scale (VAS); Temperament and Character Inventory (TCI); |
| Psychiatric morbidity and quality of life inpatients with chronic idiopathic urticaria | Ozkan 2007 | 2007 | M, 13; F, 71 | Psychiatric diagnosis in 60% of the patients. . The most frequently occurring psychiatric diagnosis was depressive disorders (40%).Seventy-one patients reported that they were not sufficiently informed about the features of the disorder. Most patients (81%) believed that their illnesses were due to stress,76% of the patients thought that their body images were disturbed in some degrees because of the illness, and 17% of the patients complained that their sexual activities were de-creased. The patients who believed that their illnesses were due to stressful events and that their body images were severely disturbed because of the illness and who complained that their sexual activities were decreased | A generic form of the General Health Questionnaire ( HRQL) questionnaire (Medical Outcomes Study 36-ItemShort-Form Health Survey [SF-36]), and the Structured Clinical Interview for DSM-IV Axis Disorders (SCID-I) |
